# Supplementary material for: Small molecules restore mutant mitochondrial DNA polymerase activity
Source: Nature. 2025 Apr 9;642(8067):501–7. doi: 10.1038/s41586-025-08856-9 (PMC12158775; doi:10.1038/s41586-025-08856-9)

nature portfolio

https://doi.org/10.1038/s41586-025-08856-9

Supplementary information

Small molecules restore mutant mitochondrial DNA polymerase activity

In the format provided by the authors and unedited

Supplementary Fig. 1

Dot blots used to determine steady state kinetics in Fig. 1d and Table 1.

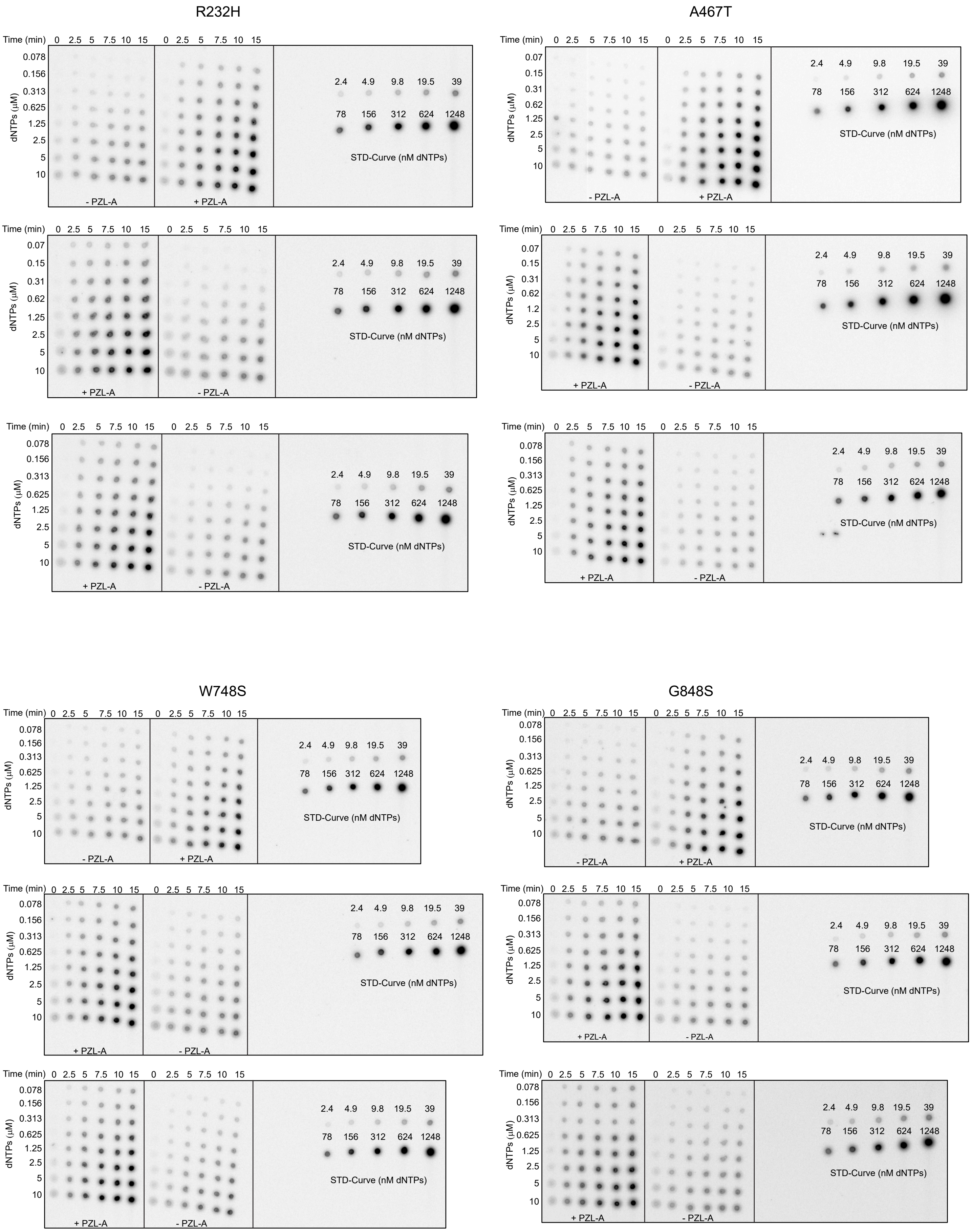

Supplementary Fig. 2

Dot blots used to determine steady state kinetics in Fig. 1d and Table 1.

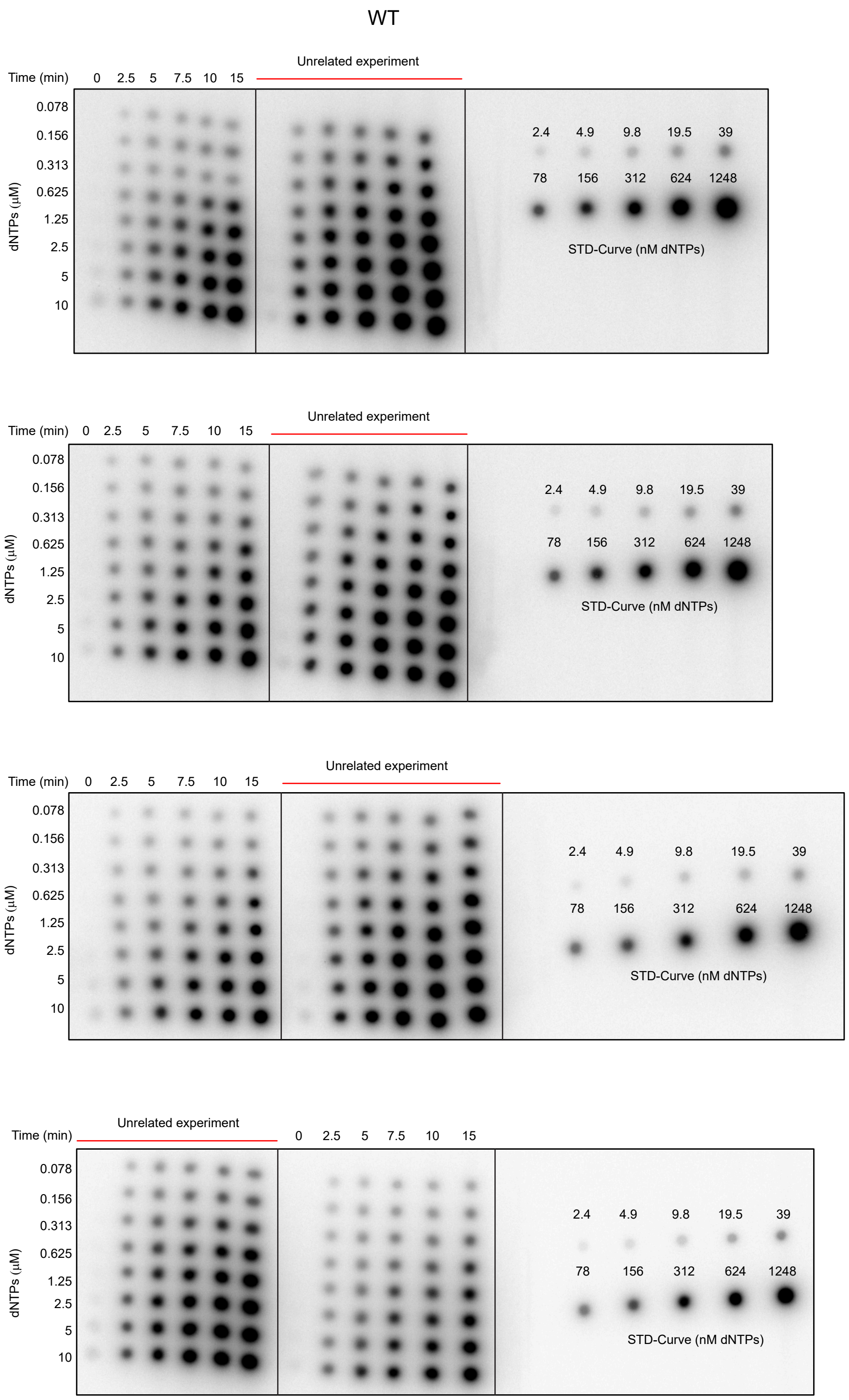

Supplementary Fig. 3

Dot blots used to determine $K_{d\_app,\ PZL\text{-}A}$ in Table 1.

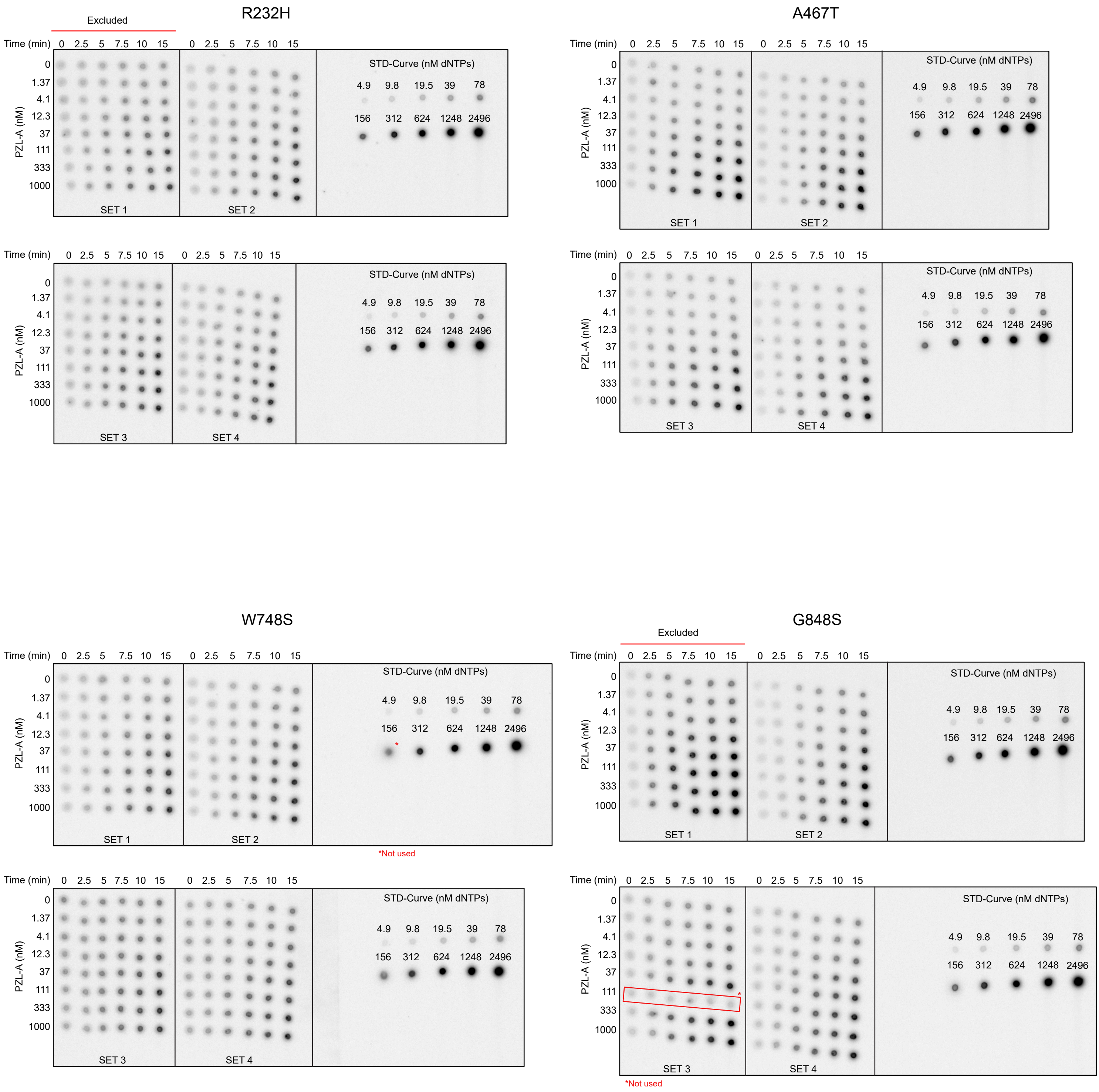

Supplementary Fig. 4

Uncropped gels and repeats used in Fig. 3a (R232H and A467T).

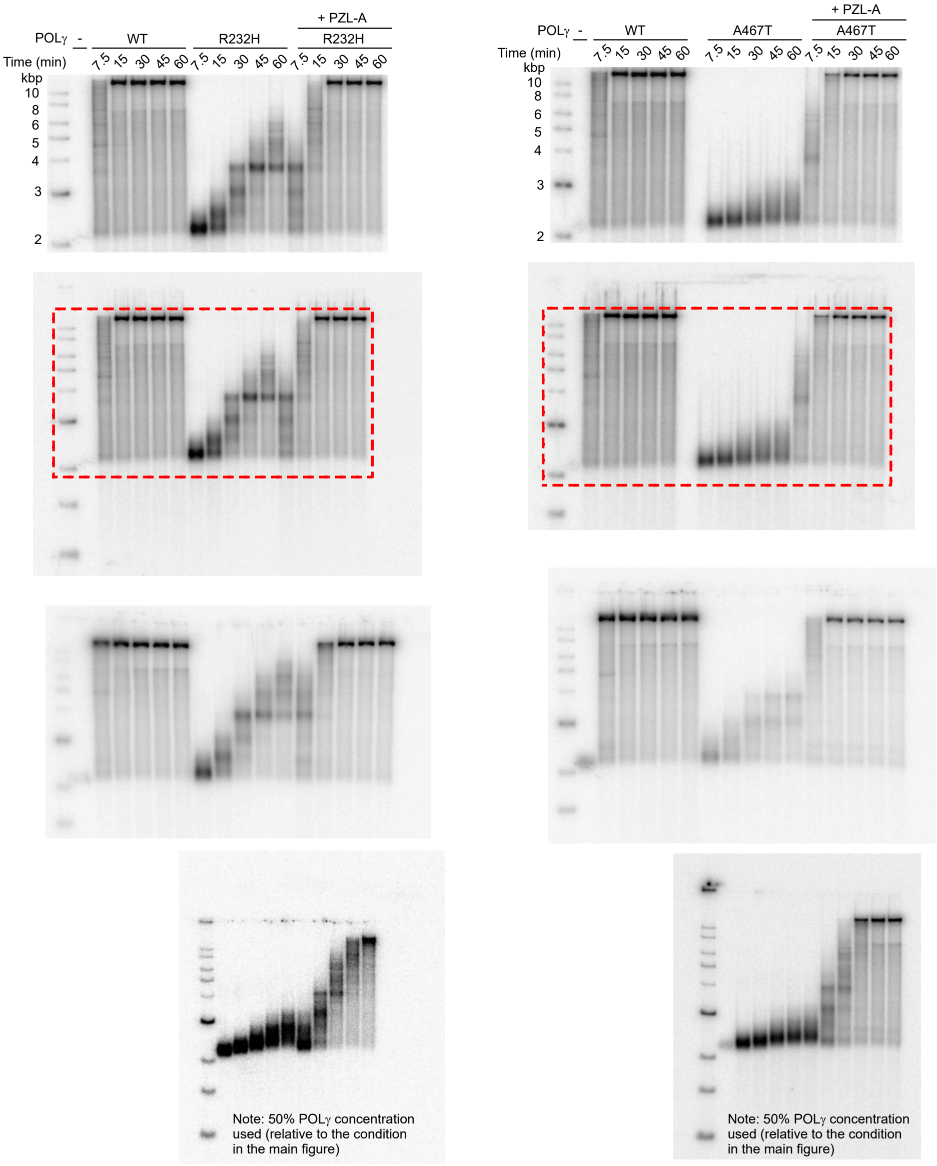

Supplementary Fig. 5

Uncropped gels and repeats used in Fig. 3a (W748S and G848S).

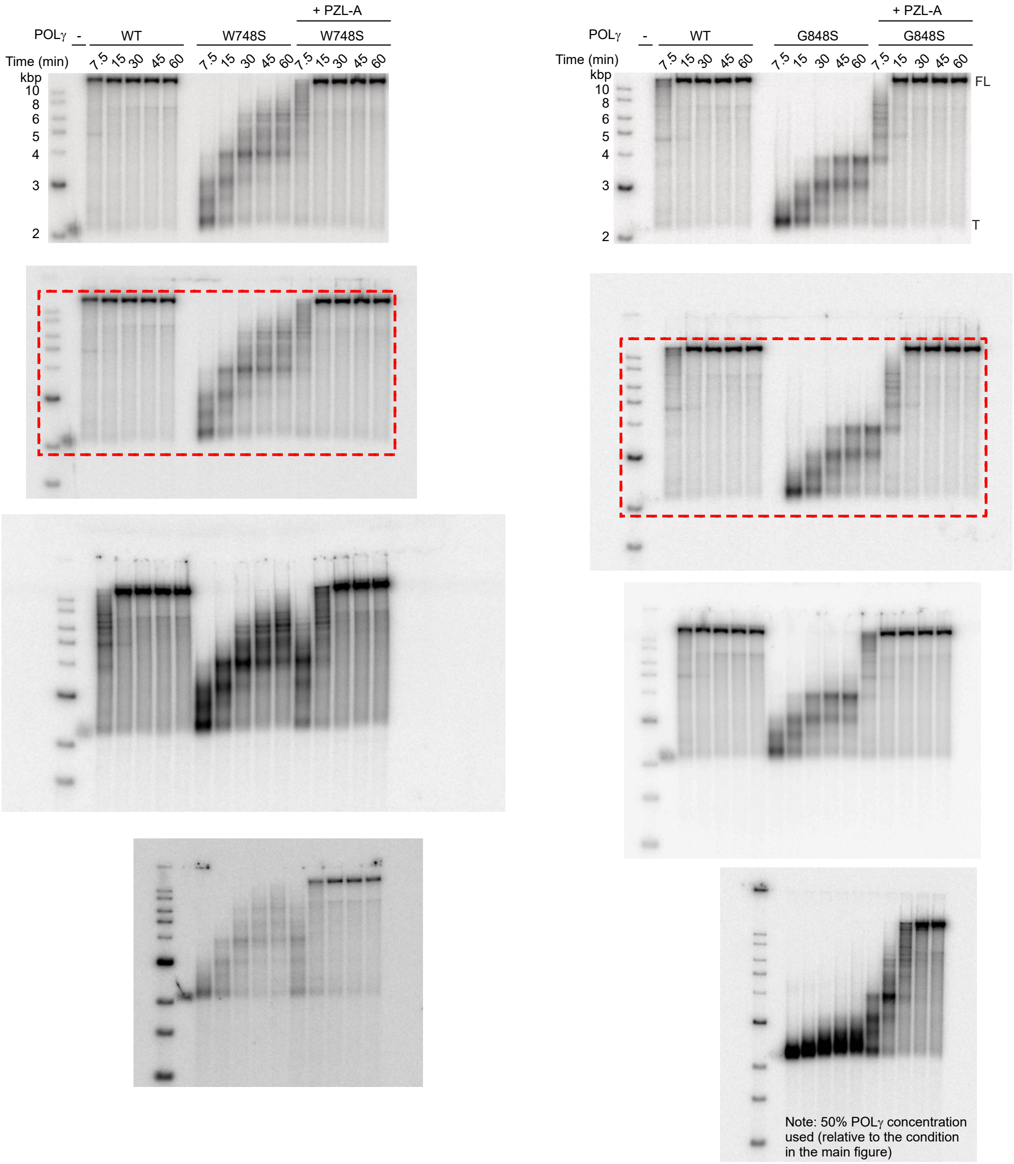

Supplementary Fig. 6

Uncropped gel used in Fig. 3c.

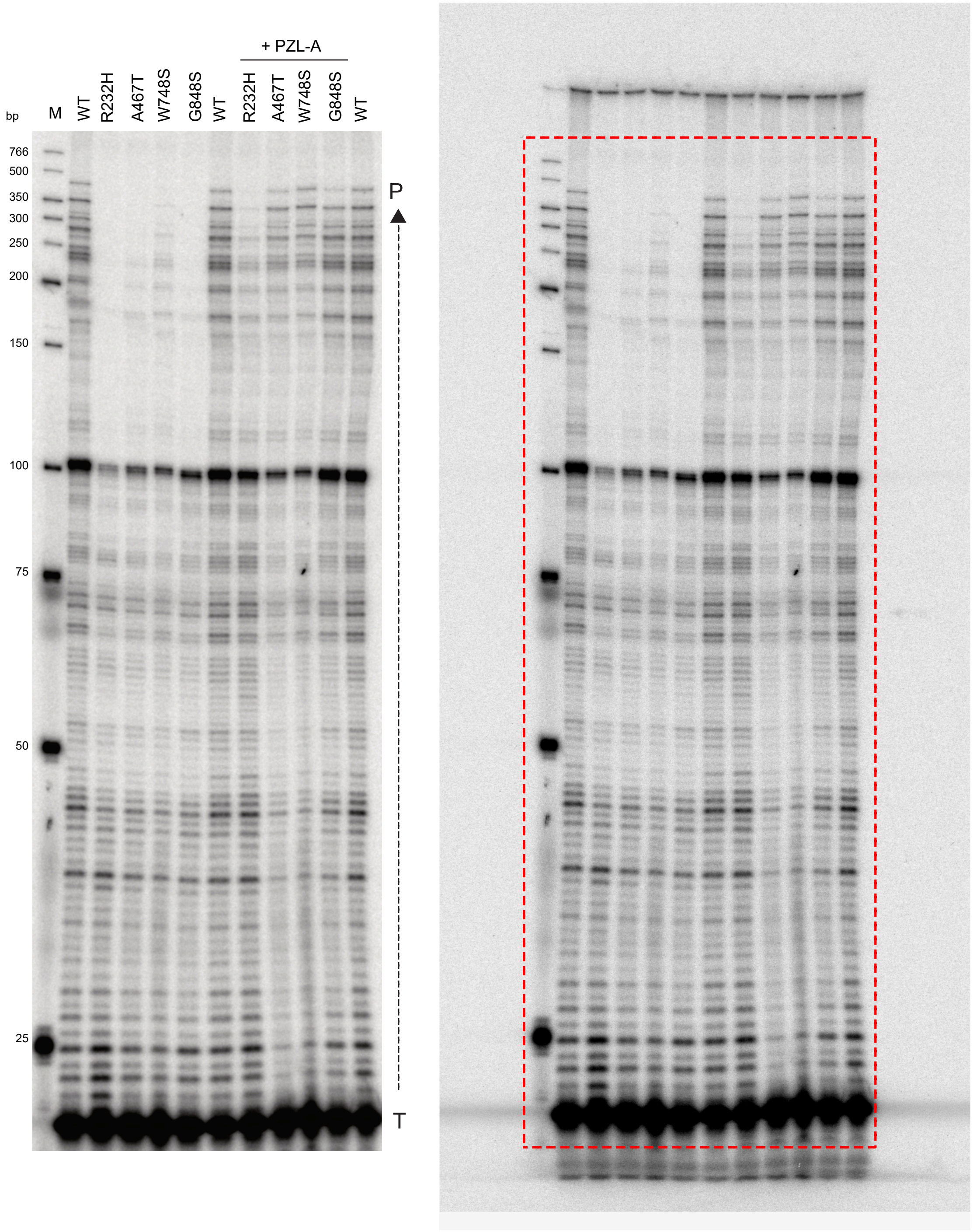

Supplementary Fig. 7

Uncropped gels and repeats used in Fig. 3e.

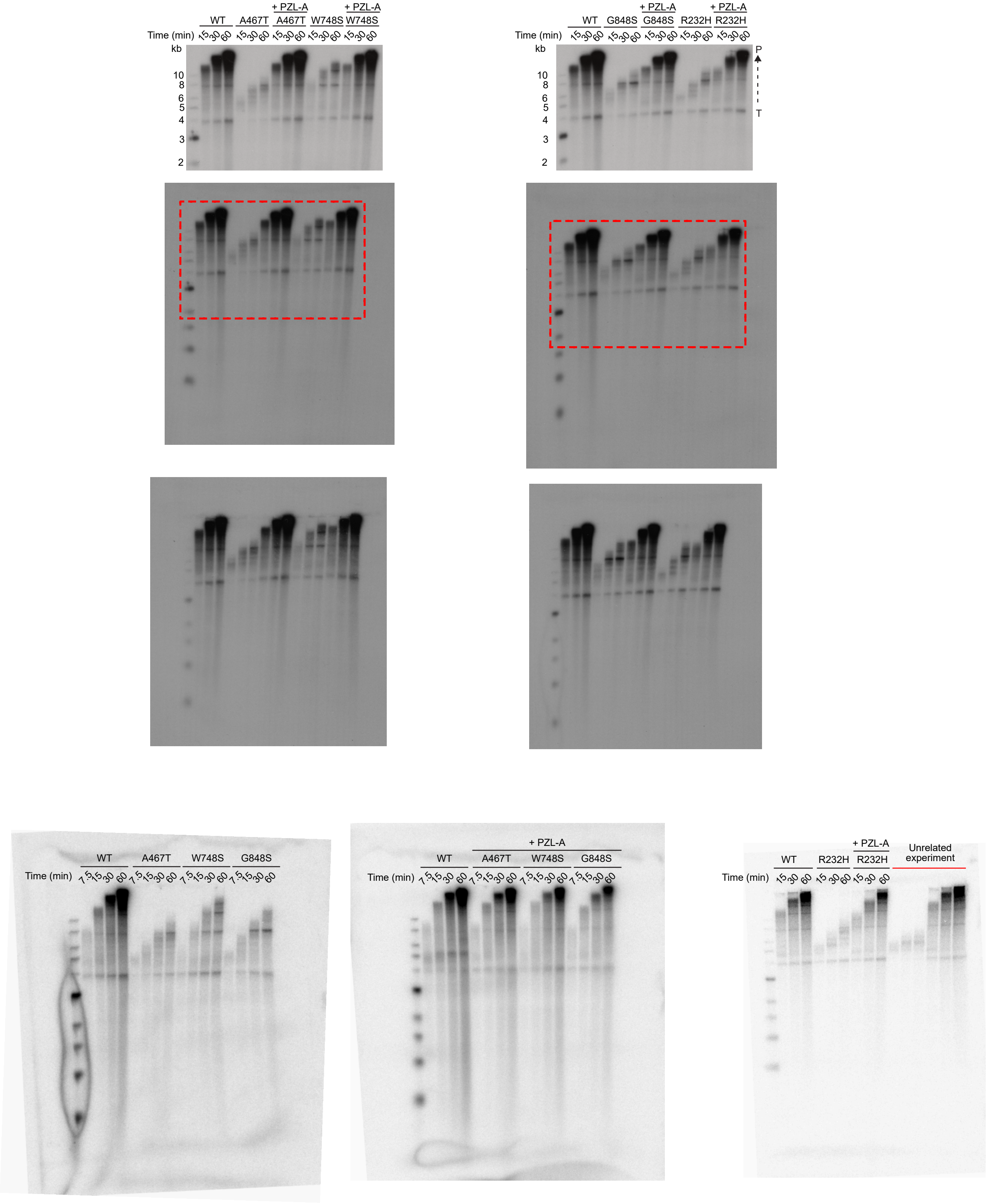

Supplementary Fig. 8

Uncropped gels and blots used in Fig. 4e–h

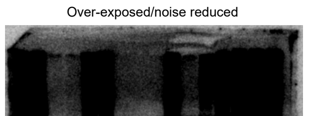

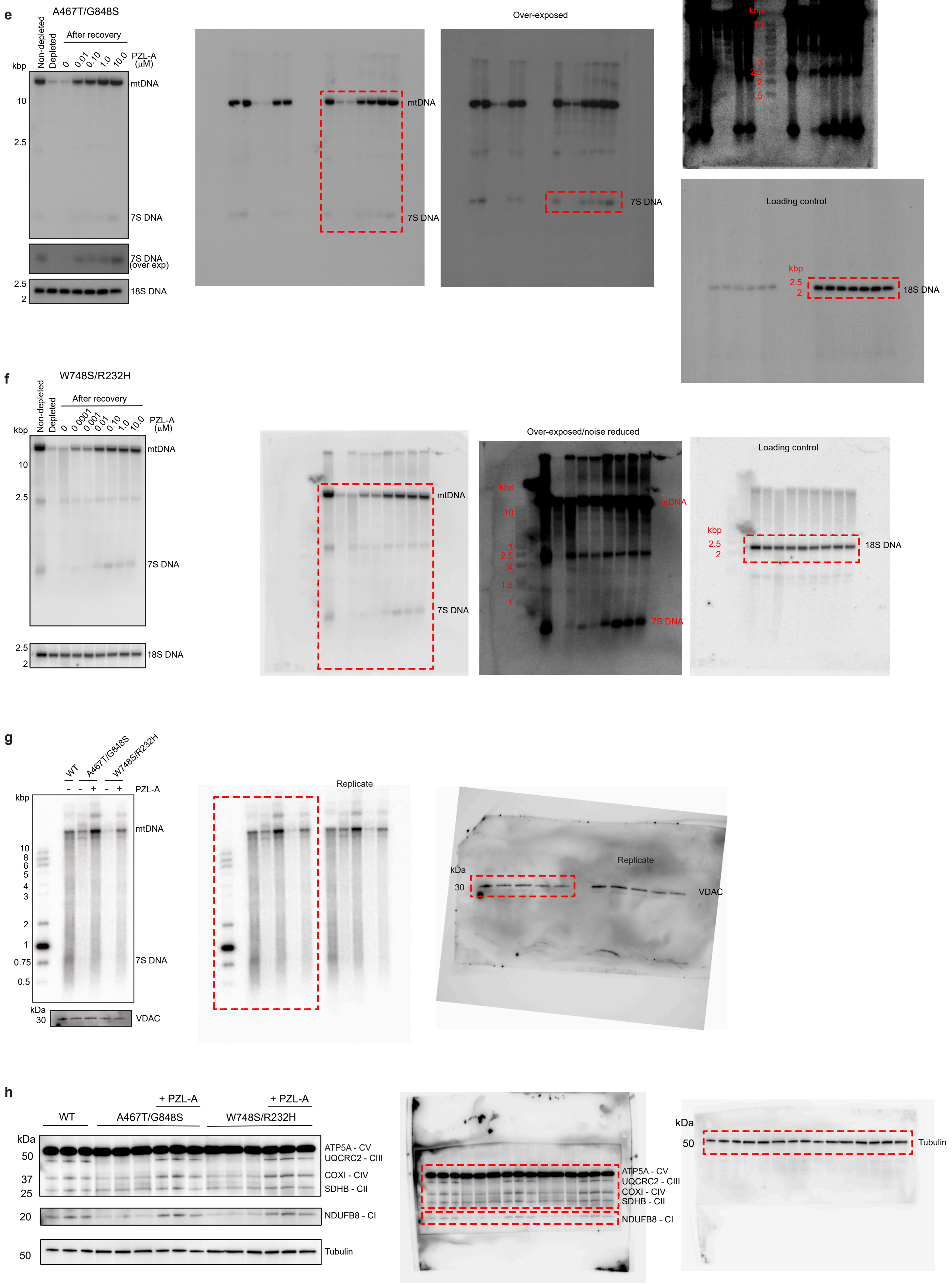

Supplementary Fig. 9

Uncropped gels and repeats used in Extended Data Fig. 1a.

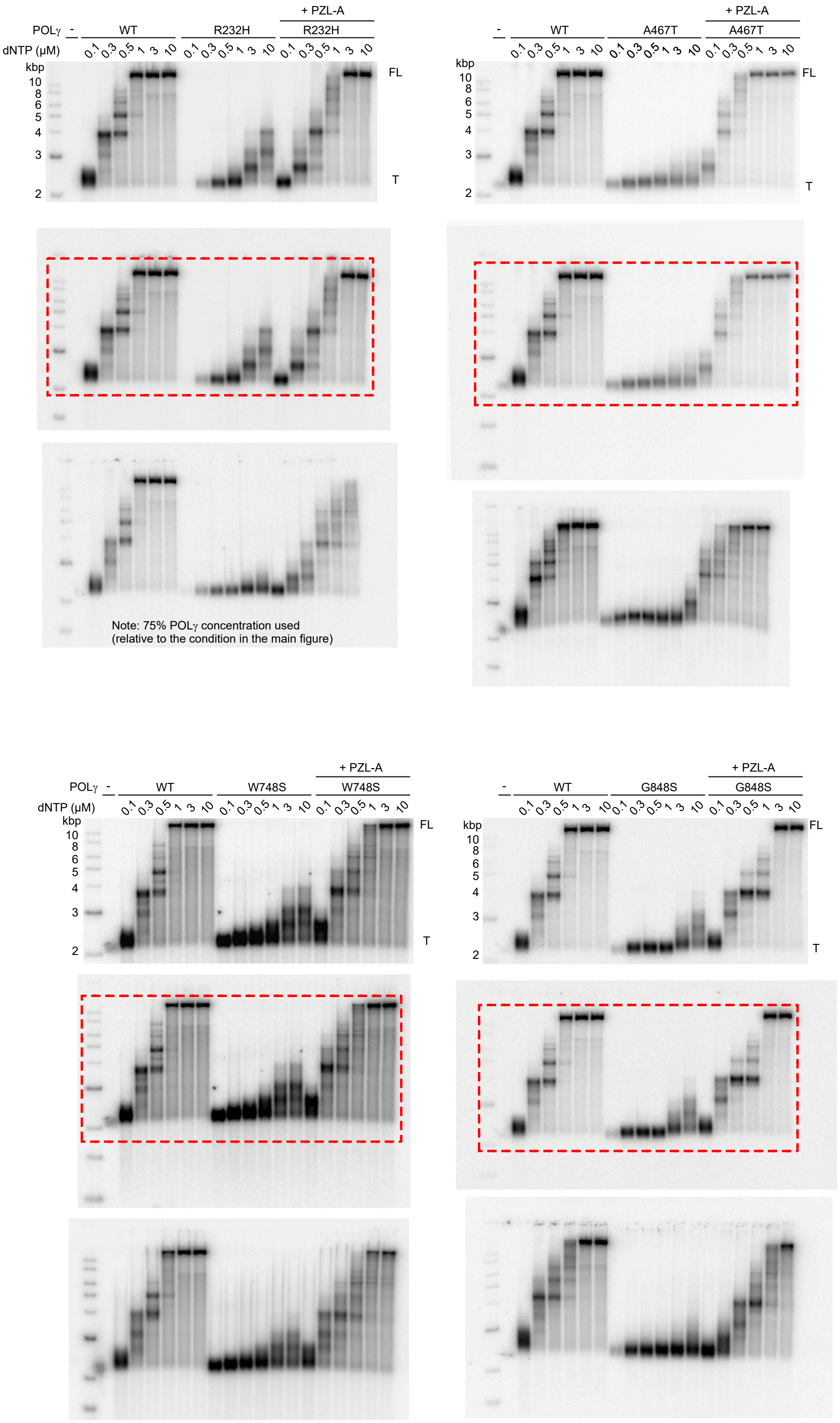

Supplementary Fig. 10

Uncropped gels used to determine active enzyme concentration in Extended Data Fig. 1b.

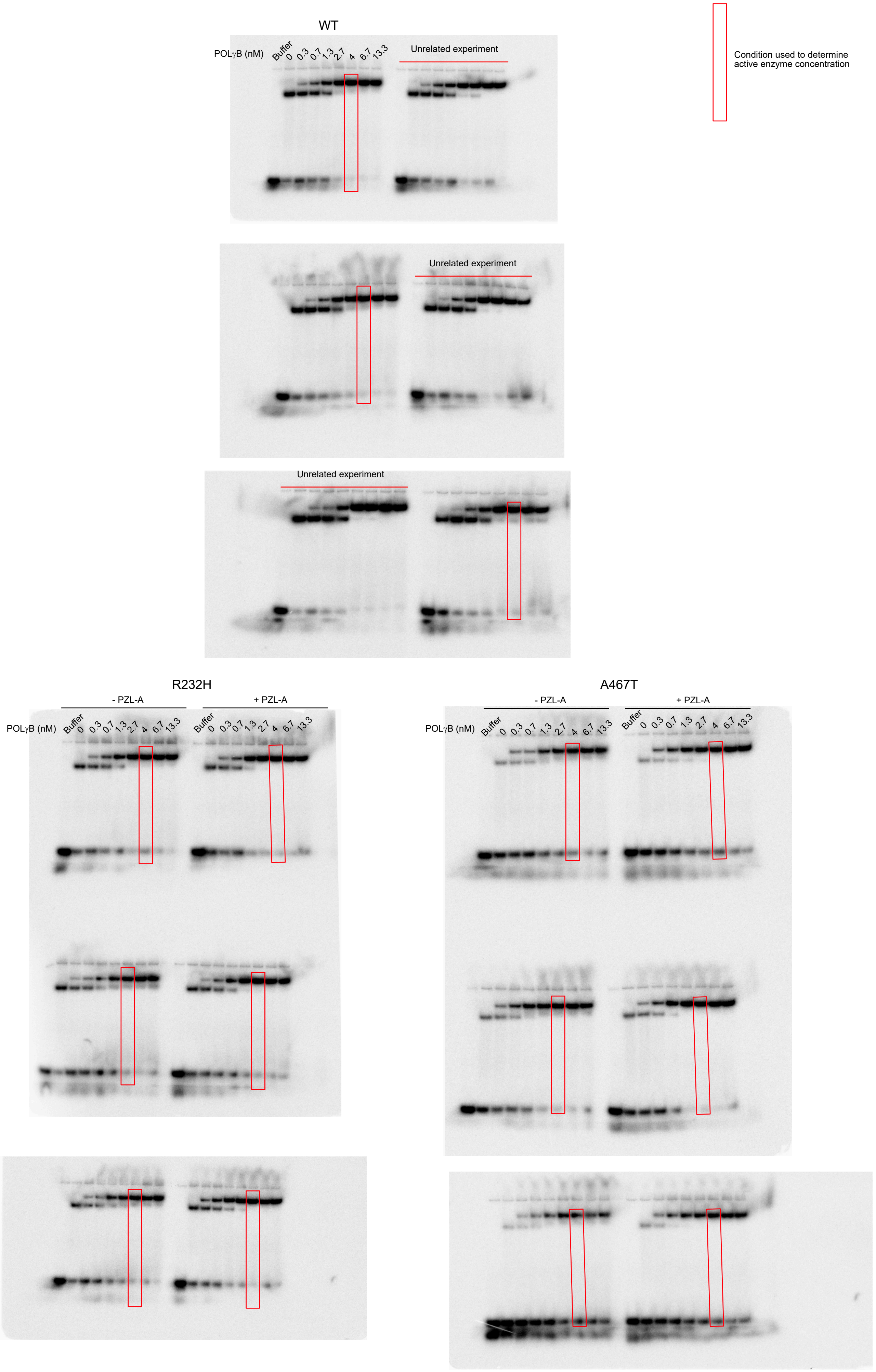

Supplementary Fig. 11

Uncropped gels used to determine active enzyme concentration in Extended Data Fig. 1b.

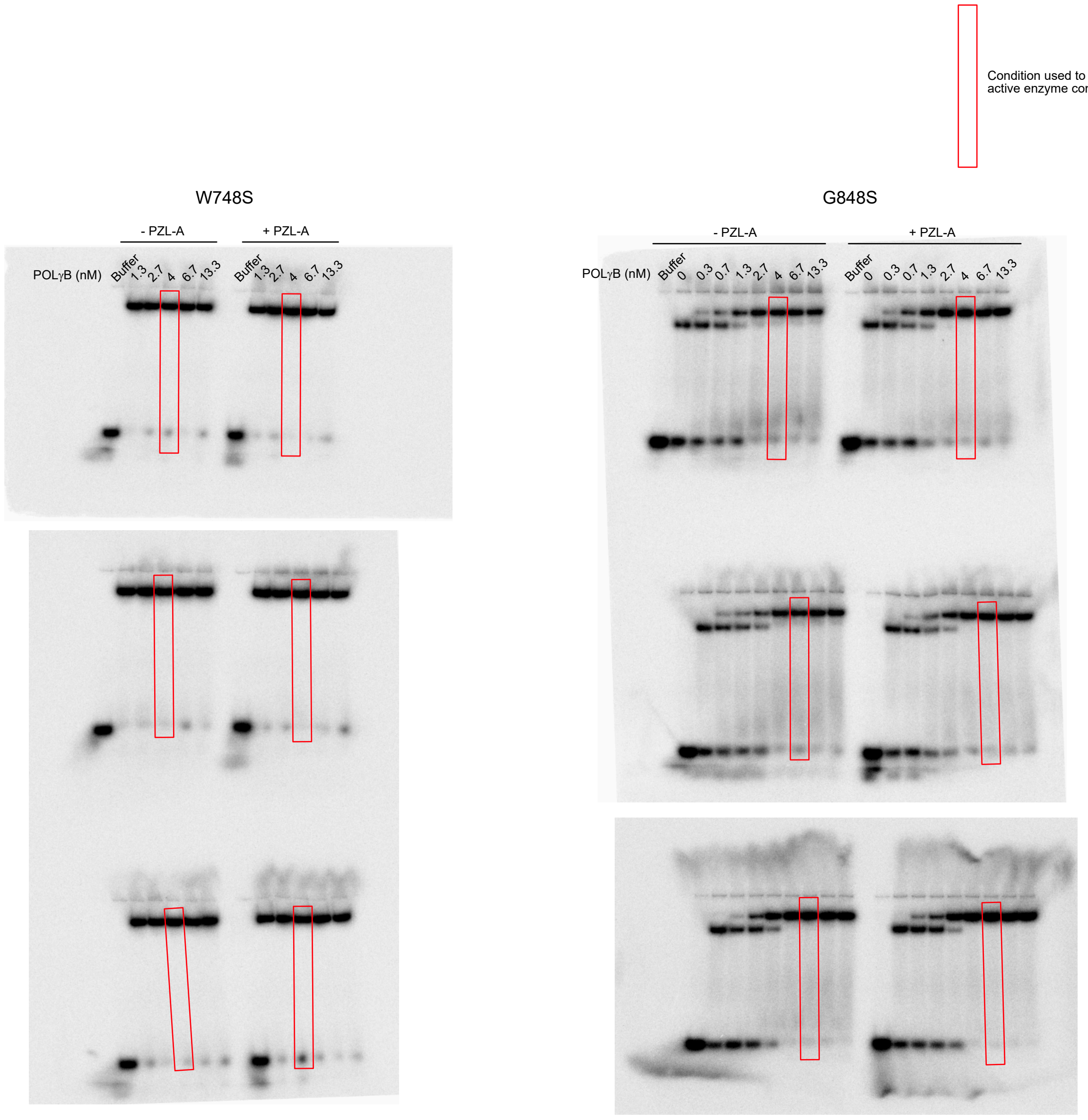

Supplementary Fig. 12

Uncropped gels and repeats used in Extended Data Fig. 1d.

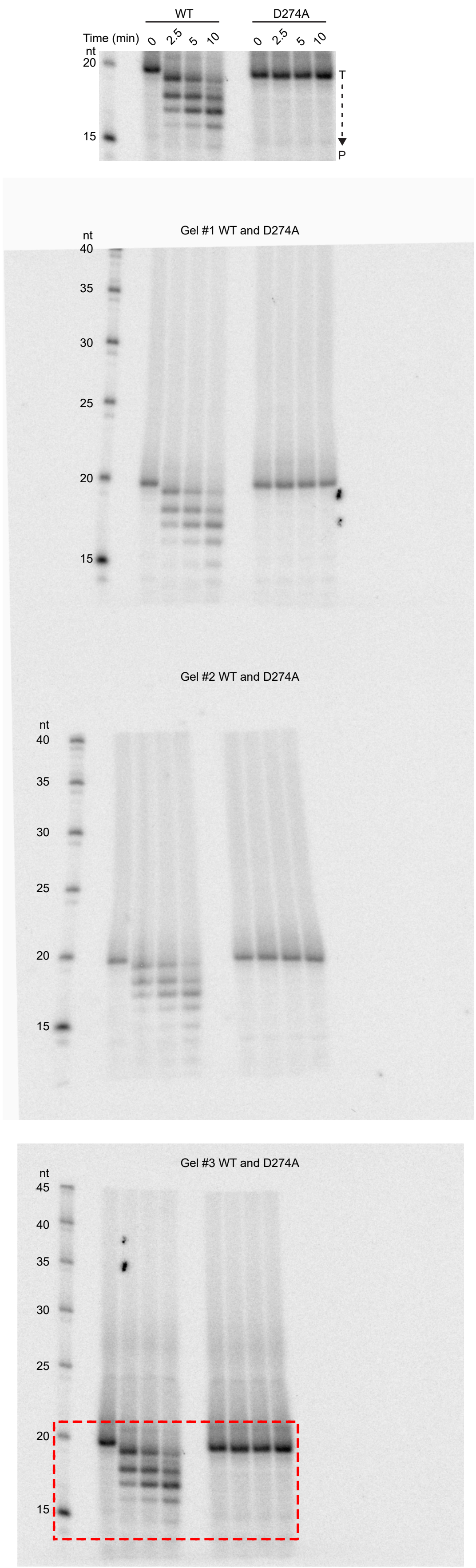

Supplementary Fig. 13

Uncropped gels and repeats used in Extended Data Fig. 1e, f.

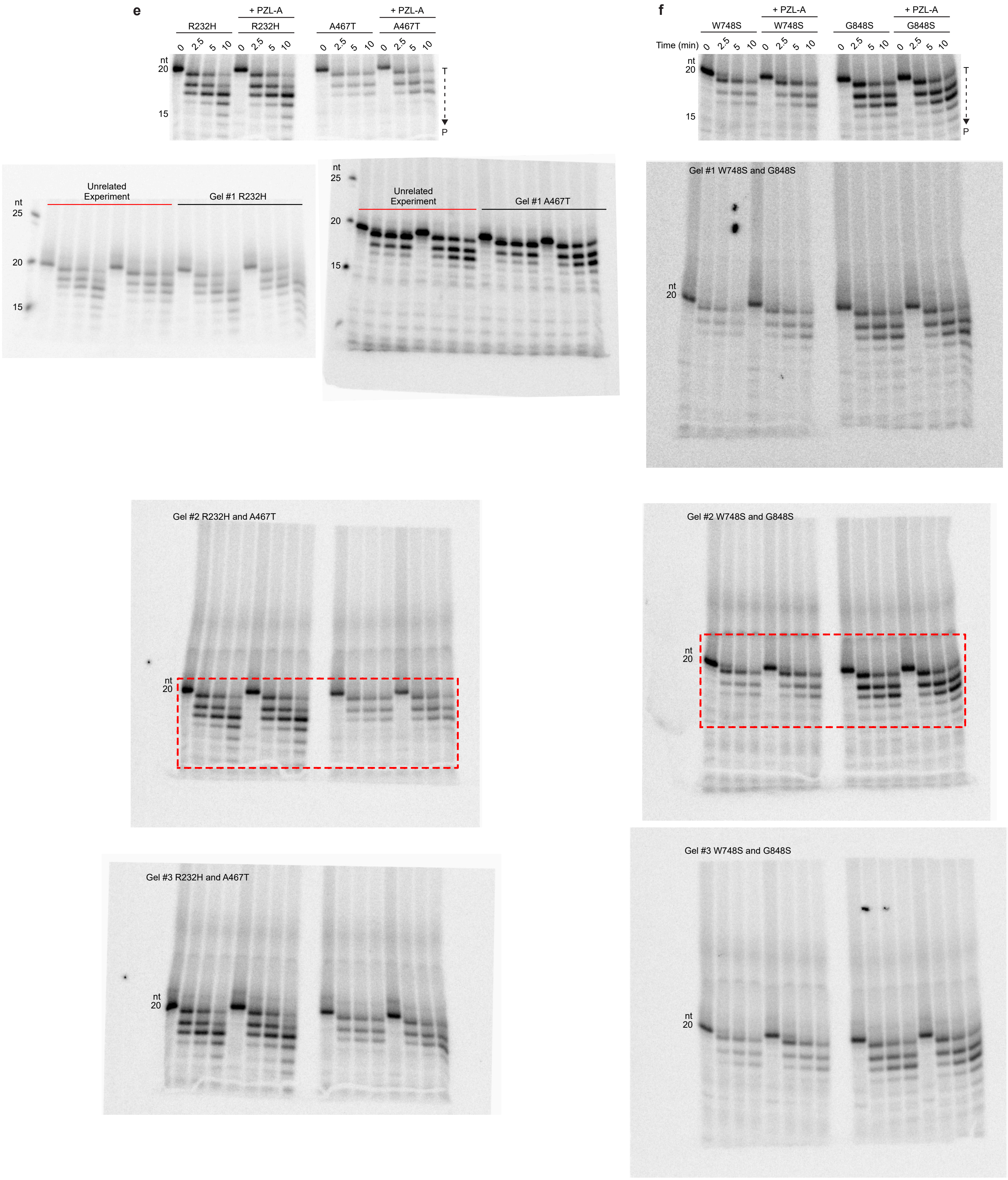

Supplementary Fig. 14

Uncropped gels used to determine K_d in Extended Data Fig. 8a, b.

WT

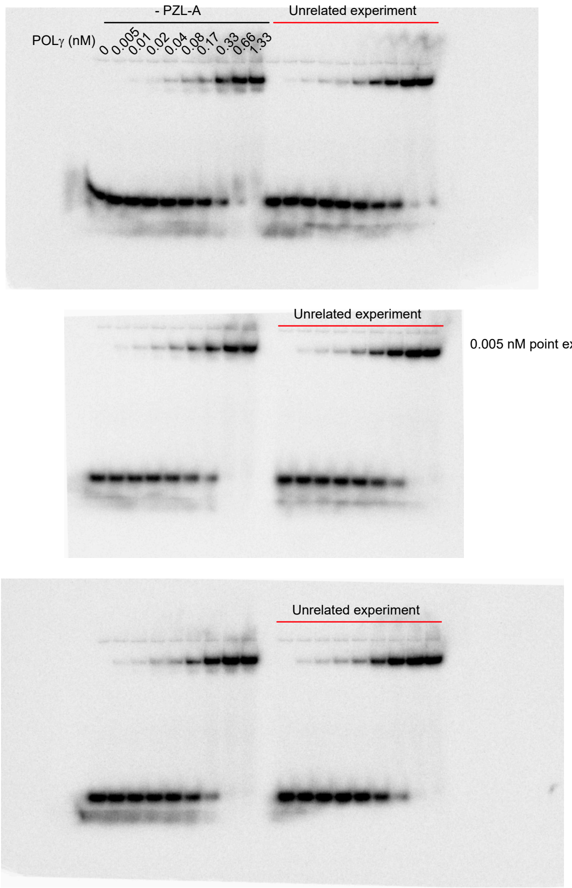

0.005 nM point excluded

0.005 nM point excluded

R232H

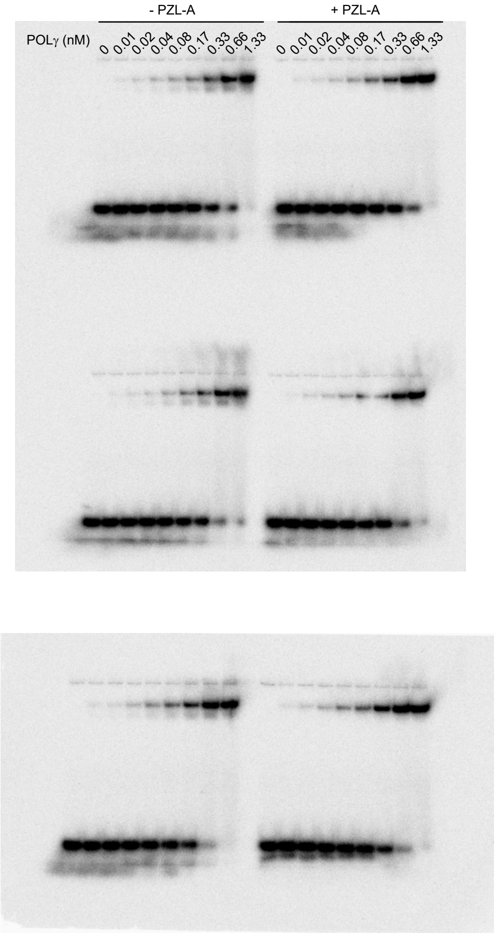

A467T

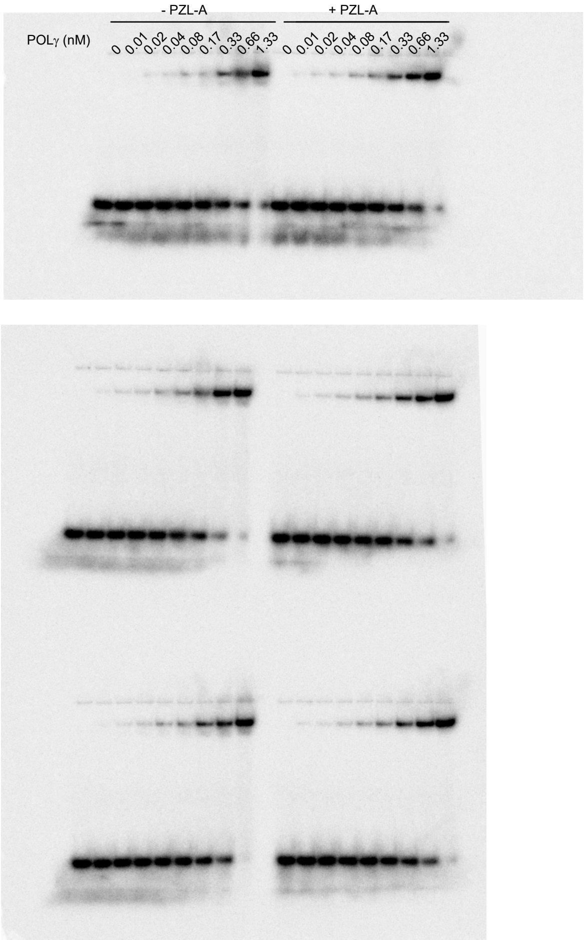

Supplementary Fig. 15

Uncropped gels used to determine K_d in Extended Data Fig. 8a, b.

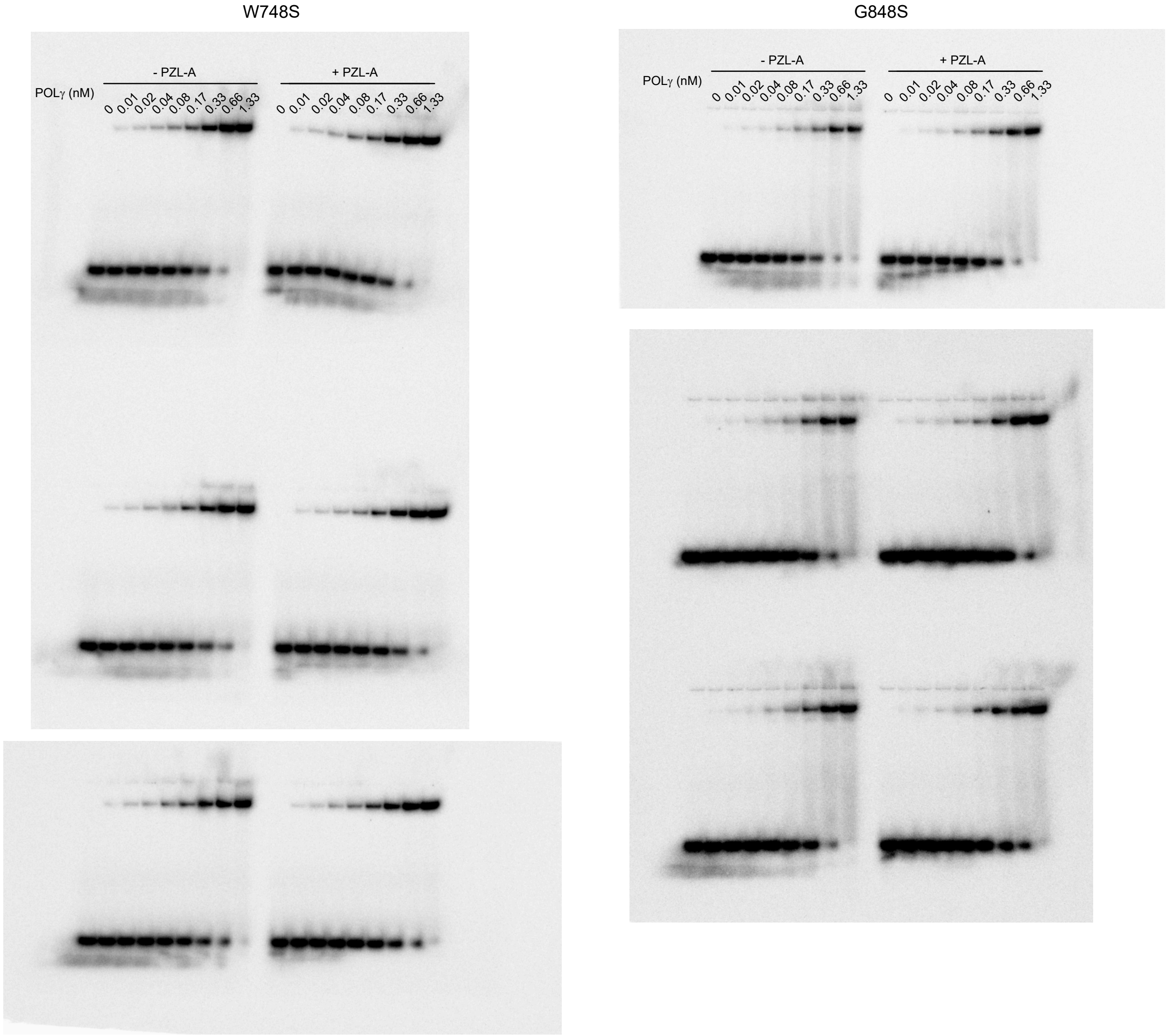

Supplementary Fig. 16

Uncropped gels used to determine k_{off} in Extended Data Fig. 8d, e.

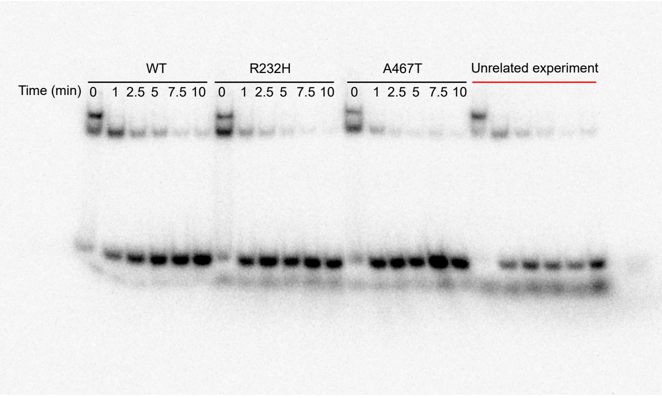

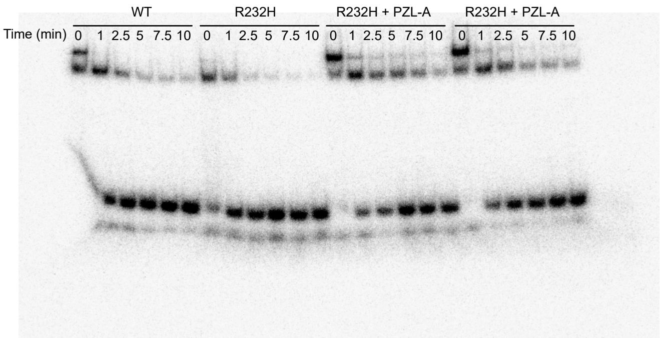

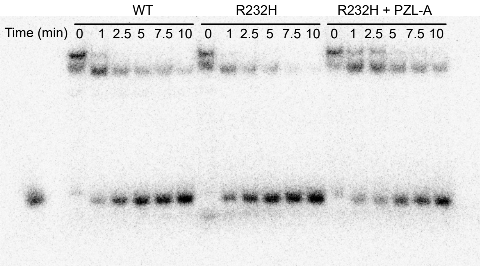

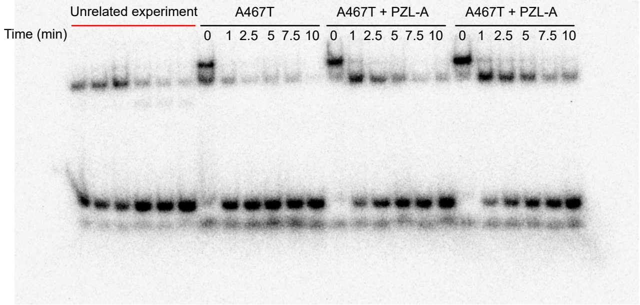

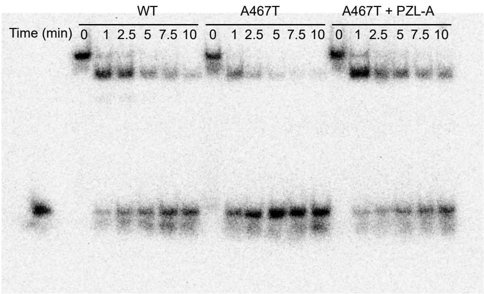

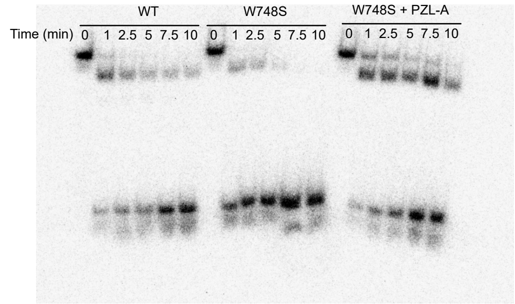

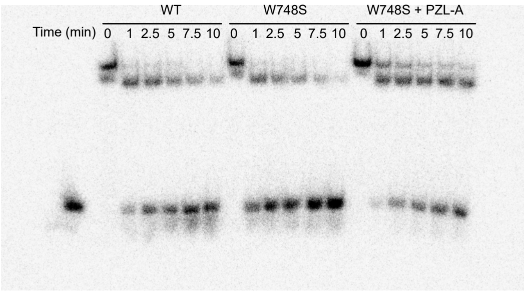

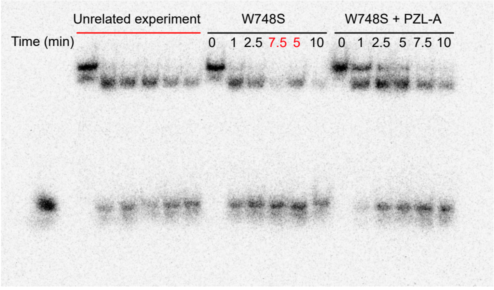

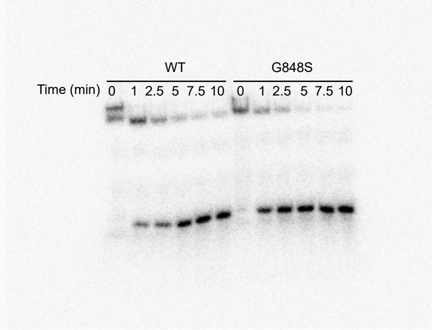

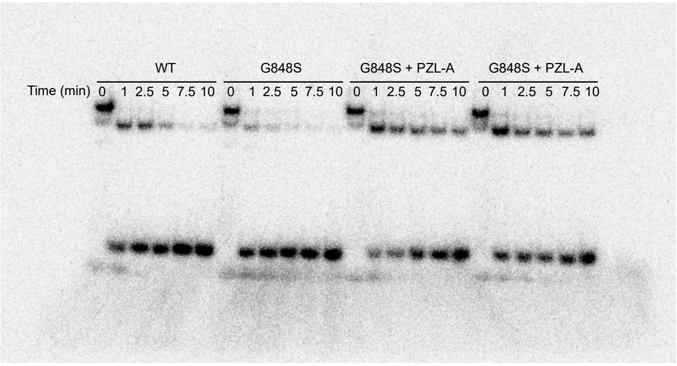

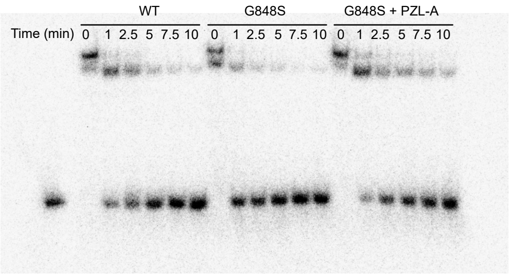

Supplementary Fig. 17

Uncropped blots used in Extended Data Fig. 9a, g.

a

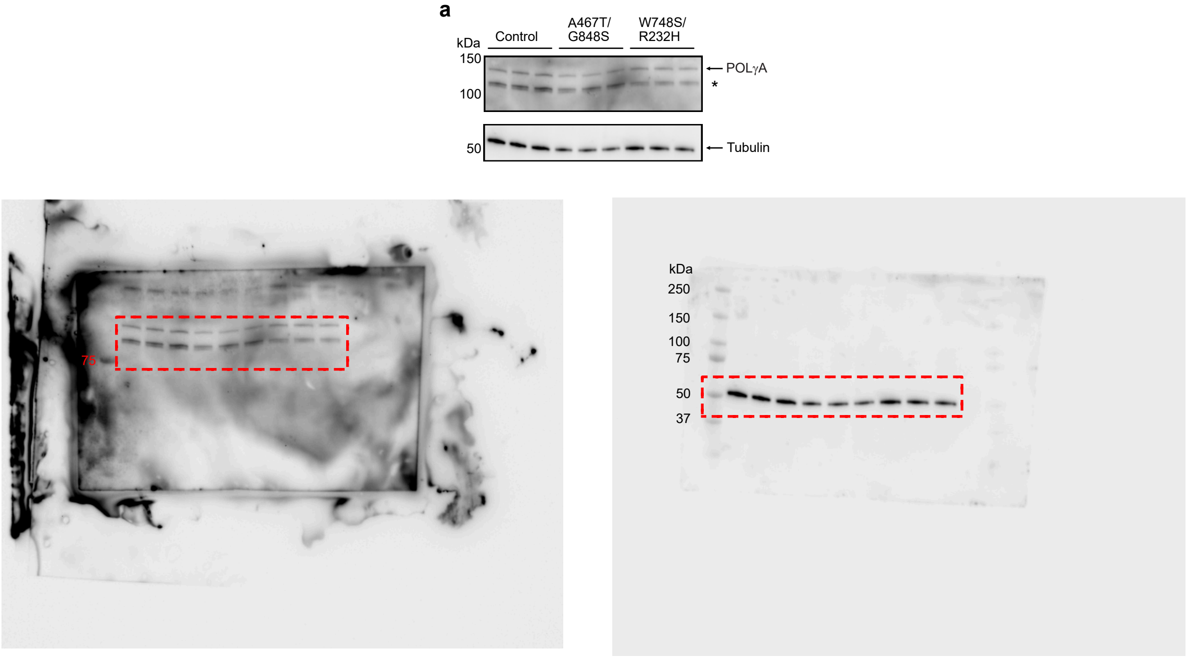

g

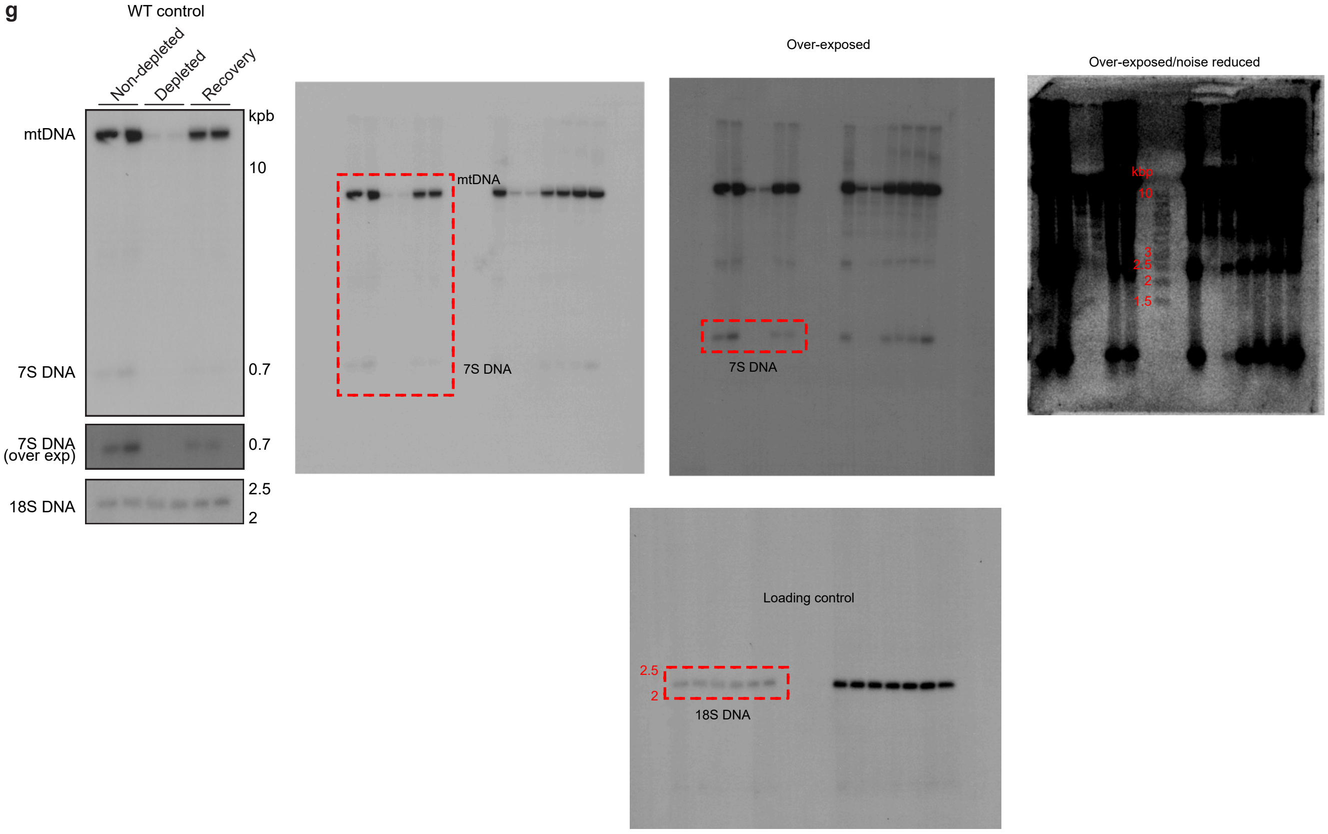

Supplement: Supplementary file 1 — This file contains Supplementary Figs. 1–17. [file 41586_2025_8856_MOESM1_ESM.pdf]
