# Supplementary material for: Small molecules restore mutant mitochondrial DNA polymerase activity
Source: Nature. 2025 Apr 9;642(8067):501–7. doi: 10.1038/s41586-025-08856-9 (PMC12158775; doi:10.1038/s41586-025-08856-9)
Supplement: Supplementary file 2 — Reporting Summary [file 41586_2025_8856_MOESM2_ESM.pdf]

nature portfolio

Corresponding author(s): Simon Giroux
Claes Gustafsson
Maria Falkenberg Gustafsson

Last updated by author(s): Feb 5, 2025

Reporting Summary

Nature Portfolio wishes to improve the reproducibility of the work that we publish. This form provides structure for consistency and transparency in reporting. For further information on Nature Portfolio policies, see our Editorial Policies and the Editorial Policy Checklist.

Statistics

For all statistical analyses, confirm that the following items are present in the figure legend, table legend, main text, or Methods section.

n/a	Confirmed	
☐	☒	The exact sample size (*n*) for each experimental group/condition, given as a discrete number and unit of measurement
☐	☒	A statement on whether measurements were taken from distinct samples or whether the same sample was measured repeatedly
☐	☒	The statistical test(s) used AND whether they are one- or two-sided<br>*Only common tests should be described solely by name; describe more complex techniques in the Methods section.*
☒	☐	A description of all covariates tested
☐	☒	A description of any assumptions or corrections, such as tests of normality and adjustment for multiple comparisons
☐	☒	A full description of the statistical parameters including central tendency (e.g. means) or other basic estimates (e.g. regression coefficient) AND variation (e.g. standard deviation) or associated estimates of uncertainty (e.g. confidence intervals)
☐	☒	For null hypothesis testing, the test statistic (e.g. *F*, *t*, *r*) with confidence intervals, effect sizes, degrees of freedom and *P* value noted<br>*Give P values as exact values whenever suitable.*
☒	☐	For Bayesian analysis, information on the choice of priors and Markov chain Monte Carlo settings
☒	☐	For hierarchical and complex designs, identification of the appropriate level for tests and full reporting of outcomes
☒	☐	Estimates of effect sizes (e.g. Cohen's *d*, Pearson's *r*), indicating how they were calculated

Our web collection on statistics for biologists contains articles on many of the points above.

Software and code

Policy information about availability of computer code

Data collection	BMG PHERAstar microtiter plate reader control software(v5.70 R6); BioRad CFX Maestro real time software(v2.3); Agilent Seahorse Wave Pro Software(v2.6)
Data analysis	In addition to above mentioned software,the following softwares were used in this study: BMG MARS data analysis software(V4.01 R2); Fujifilm Multi Gauge software(V3.1); BioRad Image lab software(v6.1);cryoSPARC (v4.3.1);DeepEMhancer(v0.14); Coot (v.0.9.8.1) and ISOLDE (v.1.4);PHENIX (v.1.20.);UCSF ChimeraX(v1.4); Graphpad Prism Software (v8.0 and 10.0);MolProbity(v4.02b-467)

For manuscripts utilizing custom algorithms or software that are central to the research but not yet described in published literature, software must be made available to editors and reviewers. We strongly encourage code deposition in a community repository (e.g. GitHub). See the Nature Portfolio guidelines for submitting code & software for further information.

Data

Policy information about availability of data

All manuscripts must include a data availability statement. This statement should provide the following information, where applicable:

- Accession codes, unique identifiers, or web links for publicly available datasets
- A description of any restrictions on data availability
- For clinical datasets or third party data, please ensure that the statement adheres to our policy

All relevant data generated and analyzed in this study are available online. Uncropped gels and raw data are provided in the supplementary information and source

data. For POLG mutations the human DNA Polymerase Gamma Mutation Database was used (https://tools.niehs.nih.gov/polg/). The atomic models and cryo-EM density maps have been deposited in the Protein Data Bank and the Electron Microscopy Data Bank under the following accession codes: G848S-PZL-A (9GGB, EMD-51326), G848S (9GGC, EMD-51327), A467T-PZL-A (9GGD, EMD-51328), A467T (9GGE, EMD-51329) and wild-type POLg (9GGF, EMD-51330). PZL-A was assigned a ligand ID (A1IK1) in the Chemical Component Dictionary.

Research involving human participants, their data, or biological material

Policy information about studies with human participants or human data. See also policy information about sex, gender (identity/presentation), and sexual orientation and race, ethnicity and racism.

Reporting on sex and gender	No sex and gender were applied in this study.
Reporting on race, ethnicity, or other socially relevant groupings	No race, ethnicity and socially relevant groupings were applied in this study.
Population characteristics	N/A
Recruitment	N/A
Ethics oversight	N/A

Note that full information on the approval of the study protocol must also be provided in the manuscript.

Field-specific reporting

Please select the one below that is the best fit for your research. If you are not sure, read the appropriate sections before making your selection.

☒ Life sciences ☐ Behavioural & social sciences ☐ Ecological, evolutionary & environmental sciences

For a reference copy of the document with all sections, see nature.com/documents/nr-reporting-summary-flat.pdf

Life sciences study design

All studies must disclose on these points even when the disclosure is negative.

Sample size	We use a sample size of n=3 in our cellular and biochemical experiments as it aligns with standard practices in the field, providing sufficient replicates to assess experimental reproducibility while balancing feasibility, resource constraints, and biological variability.
Data exclusions	No samples were excluded
Replication	All experimental data was reliably reproduced as indicated in the method section and figure legends.
Randomization	Randomization was not applied in this study because our experiments were based on biochemical and cellular assays rather than subject allocation or treatment assignment. All experimental conditions were tightly controlled by using standardized protocols, including identical cell culture conditions, reagent batches, and assay parameters, thereby minimizing potential sources of variability.
Blinding	Investigators were not blinded to experimental design and outcome. Investigators were also not blinded during data analysis. Blinding was not applied in this study because the experiments were based on biochemical and cellular assays, where measurements were obtained using objective, quantitative methods such as fluorescence-based enzymatic assays, qPCR, and cryo-EM structural analysis. These techniques provide direct and unbiased readouts, minimizing the risk of subjective bias in data interpretation.

Reporting for specific materials, systems and methods

We require information from authors about some types of materials, experimental systems and methods used in many studies. Here, indicate whether each material, system or method listed is relevant to your study. If you are not sure if a list item applies to your research, read the appropriate section before selecting a response.

Materials & experimental systems

n/a	Involved in the study
☐	☒ Antibodies
☐	☒ Eukaryotic cell lines
☒	☐ Palaeontology and archaeology
☒	☐ Animals and other organisms
☒	☐ Clinical data
☒	☐ Dual use research of concern
☒	☐ Plants

Methods

n/a	Involved in the study
☒	☐ ChIP-seq
☒	☐ Flow cytometry
☒	☐ MRI-based neuroimaging

Antibodies

Antibodies used

Total OXPHOS Rodent WB Antibody Cocktail (ab110413, Abcam); anti-tubulin (T5168,Sigma); anti-VDAC(ab14734,abcam) Anti-POLG antibody(ab128899,abcam)

Validation

- Total OXPHOS Rodent WB Antibody Cocktail (ab110413, Abcam): The Abpromise covers the use of the antibody for WB application. The antibody has been referenced in 1205 publications. https://www.abcam.com/total-oxphos-rodent-wb-antibody- cocktail-ab110413.html
- anti-tubulin (T5168, Sigma): The antibody was subjected to enhanced antibody validation and referenced in 4999 publications base on citeab.com. https://www.sigmaaldrich.com/SE/en/product/sigma/t5168?srsltid=AfmBOoolvGCftVFBNQWHaoTNKBwwUzXEvbJvMulIqanrZHxRaap_z90i
https://www.citeab.com/antibodies/2304940-t5168-monoclonal-anti-tubulin-antibody-produced-in
-anti-VDAC (ab14734,abcam): The Abpromise covers the use of the antibody for WB application. The antibody has been referenced in 555 publications.
https://www.abcam.com/en-us/products/primary-antibodies/vdac1-porin-vdac3-antibody-20b12af2-ab14734
Anti-POLG antibody(ab128899,abcam) Rabbit Monoclonal POLG antibody. Suitable for WB and reacts with Mouse, Rat, Human samples. Cited in 28 publications.
https://www.citeab.com/antibodies/761776-ab128899-recombinant-anti-polg-antibody-epr7296?des=c7bdf0f22e10ee55

Eukaryotic cell lines

Policy information about cell lines and Sex and Gender in Research

Cell line source(s)

Human skin biopsy derived primary fibroblasts were obtained from the Swedish Biobank.

Authentication

All cell lines used in this study have been verified by Eurofins Genomics STR analysis of human cell lines.

Mycoplasma contamination

Cell lines were routinely examined for mycoplasma contamination (negative).

Commonly misidentified lines
(See ICLAC register)

None

Plants

Seed stocks

N/A

Novel plant genotypes

N/A

Authentication

N/A
